# Transforming the American Academy of Microbiology for social good through scientific portfolios

**DOI:** 10.1128/mbio.00828-26

**Published:** 2026-05-05

**Authors:** Nguyen K. Nguyen, Rachel M. Burckhardt, Stefano Bertuzzi, Arturo Casadevall, Vanessa Sperandio, Jay T. Lennon

**Affiliations:** 1 Director of American Academy of Microbiology, ASM 11003, Washington, DC, USA; 2 Scientific Program Officer of American Academy of Microbiology, ASM 11003, Washington, DC, USA; 3 CEO, ASM 11003, Washington, DC, USA; 4 Past Chair of the Governors of the American Academy of Microbiology, Department of Molecular Microbiology and Immunology, Johns Hopkins School of Public Health, Baltimore, Maryland, USA; 5 Chair of the Governors of the American Academy of Microbiology, Department of Medical Microbiology & Immunology, University of Wisconsin–Madison 732057, Madison, Wisconsin, USA; 6 Chair of the Academy Climate Change & Microbes Scientific Advisory Task Force, Department of Biology, Indiana University 1772, Bloomington, Indiana, USA; Georgia Institute of Technology, Atlanta, Georgia, USA

**Keywords:** climate change, American Academy of Microbiology, scientific societies, think tank, microbial solutions, science strategy

## Abstract

As the research landscape evolves, scientific societies must adapt their programs to meet changing community needs. The American Academy of Microbiology (Academy or AAM) has recently developed a new model centered around scientific portfolios aimed at advancing its vision of becoming an effective scientific think tank. Here, we describe this transition and the process used to develop and implement a portfolio-based approach. We highlight the Climate Change and Microbes Scientific Portfolio as a case study, demonstrating its successes and its ability to guide the design of future portfolios.

## EDITORIAL

The American Academy of Microbiology (Academy) is an honorific group within the ASM. Its mission has been to recognize and promote excellence in the microbiology community. In 2021, the Academy set a new strategic plan and expanded the vision to become a scientific think tank (1). To achieve this goal, the Academy needs to consider how its programs effectively contribute to the scientific community but also to society at large. Since its inception, the Academy has been effective in bringing together leaders to develop scientific consensus and new ideas for the field through a colloquium program. For decades, this program addressed a broad range of topics, spanning graduate education, the environmental impacts of microbes on global health, microbial stewardship, and diagnostic paradigms.

Despite its many strengths, the colloquium-based model had its limitations. Notably, it restricted the Academy’s ability to engage deeply within a given topic. The colloquium program was designed to respond quickly to various scientific requests from the community rather than to support the sustained development of ideas over time. Scientific advances require time and consistent effort to refine and amplify ideas. To realize these goals and deliver social impact, a focused approach is needed. By doing so, the Academy would be in a better position to leverage the strength of other ASM programs.

Following its reform, the Academy was repositioned as an integral part of ASM (1), creating a strategic opportunity to more fully integrate science into ASM programs. Achieving this vision required sustained focus, consistency, and follow-through. This shift also created an opportunity to build long-term partnerships capable of delivering sustained impact. Although the Academy has collaborated with other societies and organizations in the past, these efforts were typically limited to a single colloquium over relatively short timeframes. Such engagements constrained the development of trust and continuity, limiting the Academy’s ability to position itself as a reliable partner and a forward-looking scientific think tank committed to advancing scientific insights and promoting excellence.

## EMBRACING A NEW PROGRAM MODEL: THE SCIENTIFIC PORTFOLIO

Recognizing both the strengths and limitations of the colloquium-based approach, the Academy Governors made a strategic decision to adopt a new model centered on scientific portfolios. At the core of this strategy is a sustained focus on a single overarching theme over a 5-year period. This theme is selected based on the scientific interests and input of the Academy fellows, with the goal of fostering an inclusive and engaging process that gives fellows a central role in shaping the Academy’s scientific direction. Selected themes must address pressing scientific needs, align with the Academy’s vision, span multiple scientific disciplines, and deliver important societal benefits. Through this process, the Academy Governors selected “Climate Change & Microbes” to be the topic of the first scientific portfolio. In the remaining sections, we outline the key principles and features of the portfolio model. With an emphasis on the Climate Change & Microbes Scientific Portfolio (the Portfolio), our goal is to provide a case study to facilitate the development of similar scientific initiatives in the future.

## DEFINING THE STRATEGY OF A SCIENTIFIC PORTFOLIO

The first step in launching a portfolio is to define its strategy and goals. For the first portfolio, the Academy identified climate science and microbiology as two major scientific fields with distinct methodologies, tools, and cultures. Although microbes are critical players in nearly all geochemical cycles, they are often neglected, forgotten, or less accounted for in the biological processes related to climate change. Thus, the knowledge gaps and the connections between the fields of climate science and microbiology required focused attention. With proper coordination and emphasis, we reasoned that enormous knowledge and resources in both fields could be leveraged to deliver faster, cheaper, and scalable climate solutions. Therefore, the Climate Change & Microbes Scientific Portfolio needed to identify an agenda that was ambitious enough to inspire scientific communities and actionable enough to guide a coherent 5-year program. It was both a challenge and an opportunity for the Academy.

After much deliberation, the Academy articulated three goals for the Climate Change & Microbes Scientific Portfolio:

1. Provide scientific leadership by strengthening the understanding of the fundamental role of microbes in climate change.
2. Inform climate-related policies and guide advocacy efforts.
3. Inspire product innovation and accelerate the development of microbe-based solutions to mitigate climate change.

**Fig 1:**
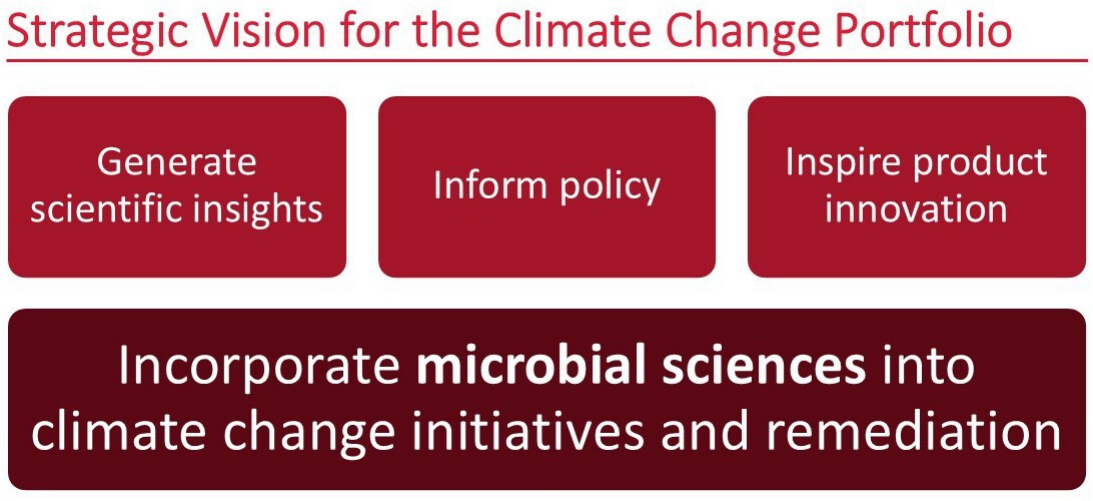
Strategic framework diagram showing three goals of the Portfolio to incorporate microbial sciences into climate change initiatives and remediation: generate scientific insights, inform policy, and inspire product innovation.

The Portfolio emphasized scientific convening, sustained partnership, and turning science into practice, with the goal of shaping research trajectories and accelerating real-world solutions. Together, these goals aimed to elevate microbial sciences within climate discourse and ensure that microbial contributions are integrated into climate solutions at regional and global scales (Fig. 1).

**Fig 2:**
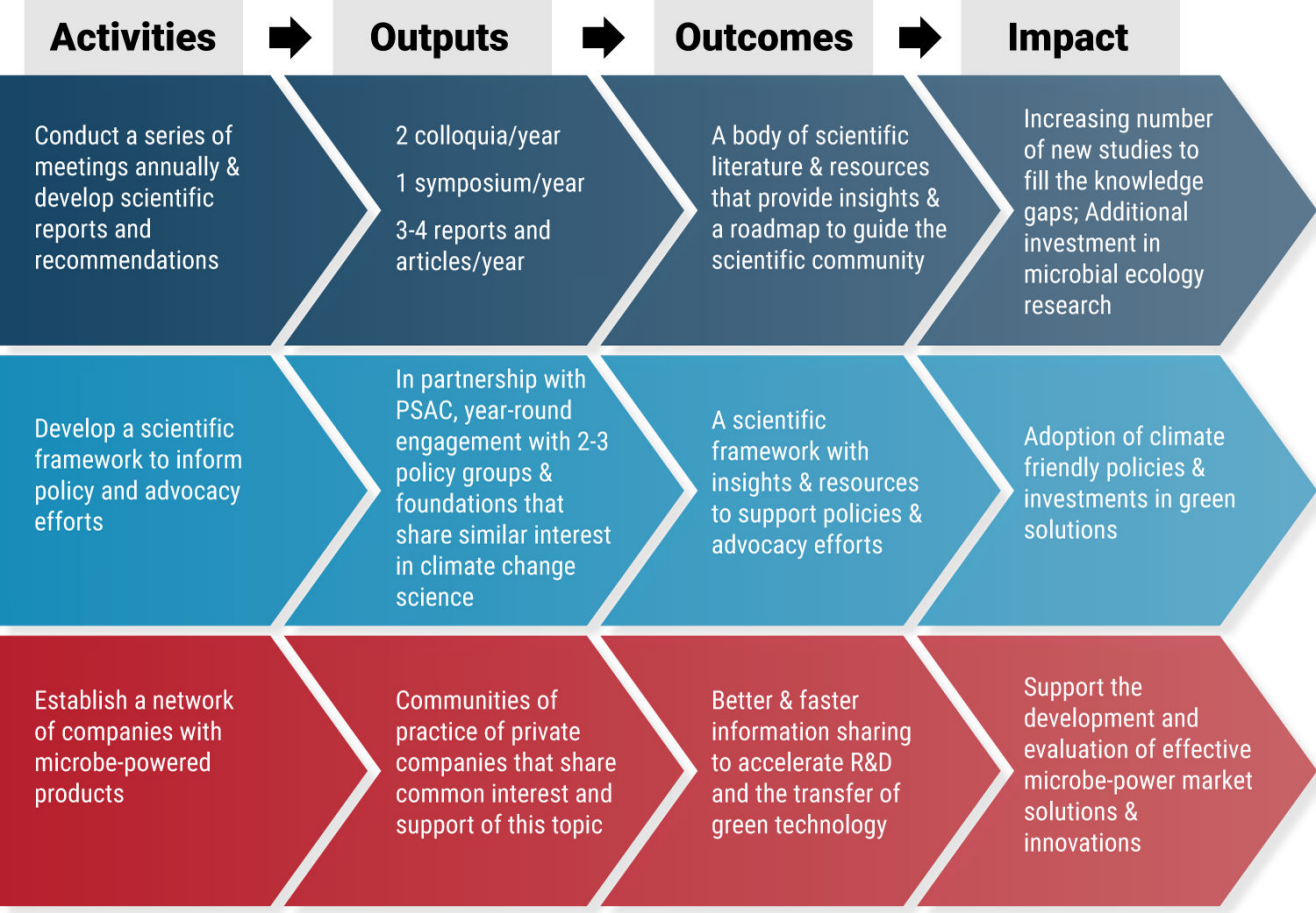
Theory of change diagram with three strategic pathways linking research activities to climate impact. Pathways include scientific convening and publications, scientific framework for policy development, and establishment of microbial technology networks.

Central to this strategy was an emphasis on building networks. The Portfolio sought to engage microbiologists and other scientific experts to also represent diverse sectors, including industry, government, think tanks, and scientific foundations. Partnership with these stakeholders played an important role in the successful implementation of the Portfolio. Figure 2 shows the theory of change, in which all partners work together toward the Portfolio’s strategic goals.

## MEASURES OF SUCCESS

From the beginning, the Academy defined success metrics to ensure the Portfolio delivered meaningful outcomes. The measurement framework detailed three criteria:

1. Advance scientific understanding: a successful portfolio must enable the Academy to fulfill its important role as a convener of great minds and as a synthesizer of the knowledge to produce authoritative reports to educate, inspire, and set the direction for the field.
2. Increase awareness and interest: a successful portfolio needs to elevate recognition of the fundamental roles of microbes—both within microbiology and across other disciplines, as well as among policymakers, funders, and the public.
3. Mobilize the scientific community for further actions: a successful portfolio must catalyze a strong level of interest within the microbiology community, both in the United States and globally, and empower other like-minded initiatives.

The intention of the Portfolio is to serve as a catalyst to help accelerate research and applications of microbial solutions.

## IDENTIFYING PORTFOLIO FOCUS AREAS

To guide this interdisciplinary work of the Climate Change & Microbes Portfolio, the Academy convened a Scientific Advisory Task Force. Members were selected based on their scientific expertise, professional standing, and interest in shaping the future of microbial climate research. The Task Force, chaired by Academy Governor Dr. Jay Lennon, worked in close collaboration with Academy leadership and staff to define scientific priorities and shape major initiatives. The Task Force members also participated in implementing these activities throughout the portfolio.

Through extensive meetings and discussions, the Task Force identified three scientific focus areas:

- Health, microbes, and the environment: understand infectious diseases through the lens of global change, especially emerging and re-emerging infectious microbes that are induced by climate change.
- Microbial diversity: cascading effects of climate change: explore novel interventions to reduce exposure to harmful microbes, decrease degradation of environments that host beneficial microbes, and maintain ecosystems productivity in response to a changing environment.
- Microbial controls on greenhouse gases: understand the role of microbes in planetary carbon atmospheric flows and promote their utilization in bioprocesses to reduce emissions, in particular methane in managed landscapes*.*

These scientific focus areas were integrated with the Portfolio’s strategies and used to inform decisions, priorities, and the selection of scientific programs and activities.

## OUTCOMES

The Climate Change Scientific Portfolio successfully met these criteria. Over the 5-year period, the Academy hosted five colloquia and four scientific sessions at non-ASM meetings (Table 1). Experts from a range of disciplines, including agriculture, animal science, biogeochemistry, climate science, climate modeling, ecology and evolution, engineering, hydrology, infectious disease, public health, and healthcare management, were engaged by the Academy (2–7). Experts from multiple sectors were also included in these meetings, including those working in policy, government, national laboratories, industry, and other non-profits. This format allowed novel ideas to arise. For example, the burgeoning field of “disaster microbiology” was directly conceived from a 2021 colloquium (8, 9). Bringing together diverse stakeholders allowed the Academy to hear their needs and synthesize their challenges into cohesive, strategic plans for future research opportunities. In total, these scientific convenings led to the development of reports that have been widely read and have been used to guide funding and research priorities (2–7).

**TABLE 1:** Major outcomes of the Climate Change & Microbes Scientific Portfolio 2021–2026

Major activities	Impacts
Advance scientific understanding	
Six published reports^*a*^	Over 19,500 pageviews since April 2022.
Five colloquia hosted	Over 120 scientists participated from diverse disciplines.
Thirteen scientific manuscripts^*a*^	Publications in and outside of traditional microbiology journals to reach a broader audience.
Increase awareness and interest	
Monthly newsletter	Over 530 recipients (average 46% open rate, 12% click rate).
Five articles and three webpages for science-interested public	More than 30,000 pageviews since April 2022.
Mobilize the scientific community for action	
Partnered with more than 12 other organizations	Developed or deepened partnerships with more than 12 other microbiology or science organizations. These partnerships resulted in multiple publications, at least four scientific sessions during partner annual meetings.

^*a*^ See Box 1 for the full list of publications.

The scientific outputs of the Climate Change Portfolio have catalyzed global engagement of microbiologists on this topic. In 2023, inspired by the work of the Portfolio, the International Union of Microbiological Societies (IUMS) partnered with the Academy to assemble a Scientific Advisory Group of global experts to identify the most promising microbial innovations for climate mitigation and adaptation. This group integrated scientific, economic, engineering, and policy dimensions to evaluate feasibility, impact potential, and implementation strategies (6, 10, 11). These efforts, along with others, led to the development of the first global strategy to define a roadmap for the microbiology scientific societies to work together to advance the research agenda and application of microbes to address climate change (12, 13). This strategy aims to unify scientific societies globally, including the Federation of European Microbiological Societies (FEMS), Applied Microbiology International (AMI), International Society for Microbial Ecology (ISME), IUMS, and others, around shared objectives, strengthen the research ecosystem, and ensure microbial sciences are fully represented in climate discussions (14). This is the first step to building an international coalition to ensure the microbiology community is unified in direction and speaks in one voice toward the shared goals.

## REFLECTIONS AND LOOKING FORWARD

The scientific portfolio model is a strategic move by the Academy toward its vision of fundamentally impacting microbial sciences and providing guidance and advice to leadership. In the past 5 years, the Academy has demonstrated how the scientific portfolio approach has resulted in a high level of productivity. This approach fulfilled the strategic goal set by the Academy leadership and the request from the ASM Board of Directors. The scientific portfolio approach has become a model of how science can be incorporated into the functions of a scientific society to promote scientific excellence, innovation, and societal good.

Reflecting on the successes of the Climate Change & Microbes Scientific Portfolio, the Academy’s inaugural portfolio, one important factor that contributed to its success was the tremendous support, strong capabilities, and established infrastructure provided by ASM. The portfolio requires personnel, expertise, and partnerships with other internal ASM departments, such as Journals, Meetings, Policy, Education, etc. These internal partnerships, along with ASM’s financial backing and reputation, were the prerequisites for the portfolio’s success. In other words, the portfolio was ASM’s investments toward its mission to promote and advance microbial sciences, and the Academy was at the forefront to fulfill that vision.

The portfolio has strengthened the partnerships between ASM and our organizational partners. AGU has been a key partner in many of the Academy colloquia. The Portfolio provides the platform for societies, such as the Association for the Sciences of Limnology and Oceanography (ASLO), Soil Science Society of America (SSSA), and American Society of Tropical Medicine and Hygiene (ASTMH) to partner with the Academy and ASM for the first time on a scientific activity. Other microbiological societies are working with ASM toward a shared scientific objective. Moreover, many funding agencies, such as the Burroughs Wellcome Fund, Moore Foundation, Wellcome Trust, and Schmidt Sciences have trusted the Portfolio with their support and benefited from the work of the Portfolio.

Besides the scientific accomplishments, the Portfolio has already made progress towards big goals. Through communication efforts, the Portfolio helped increase public awareness and influence policies. However, 5 years is a short period to shift those opinions. More time, strategies, and activities would be needed. Thus, the Portfolio has established a foundation and network that ASM and other societies can further develop toward the broader goals. Future portfolios should take this into consideration in their design.

Starting in 2026, the Academy will embark on the journey to establish a new portfolio focused on Sustainability and Microbes. This portfolio will build on a large body of research on how microbes can contribute to better human life, protect ecosystems, and promote prosperity (15). Building on the experience, processes, and capabilities acquired from the first portfolio, the Sustainability Scientific Portfolio is expected to greatly benefit from that knowledge and gain strong momentum within the microbiology community. The Academy has an opportunity to leverage the lessons learned and newly acquired capabilities to further the vision and bring more value to the Academy and ASM in the coming years.

The efforts and gains from the Climate Change & Microbes Scientific Portfolio will continue. ASM has recently reorganized itself around three scientific units focused on different but integrated scientific domains with the goal of empowering the whole scientific community in setting its priorities for science. This offers an ideal opportunity for the Academy to transition its innovative incubator activities on climate change to the ASM Applied Environmental Microbiology (AEM) Scientific Unit, which will carry forward the attention and the strategy within the scientific community. Together with the other two scientific units, Health and Mechanism Discovery, the AEM unit is the new ASM mechanism to deliver ASM’s Strategic Roadmap. With the goal of supporting and connecting experts in microbial sciences and developing innovative solutions for real-world applications, the AEM unit will bring broader expertise and resources to scale up the efforts started by the Academy. With one portfolio expanded and another portfolio started, the Academy enters the next phase to further test its strategy and support ASM. The excitement is high, the opportunities are many, and the path ahead is bright.

**Box 1**. **Journal articles and reports associated with the Climate Change and Microbes Scientific Portfolio**

- Our health, our action, our planet—a call to action for microbiologists to engage in climate research (2021, *mBio*)
- Microbes and climate change: a research prospectus for the future (2022, *mBio*)
- Microbes and climate change - science, people & impacts (2022, colloquium report)
- Microbiology and climate change: a transdisciplinary imperative (2023, *mBio*)
- Improved scientific knowledge of methanogenesis and methanotrophy needed to slow climate change during the next 30 years (2023, *mBio*)
- Microbes in models: integrating microbes into earth system models for understanding climate change (2023, colloquium report)
- Role of microbes in mediating methane emissions (2023, colloquium report)
- Microbial solutions must be deployed against climate catastrophe (2024, *Nature Communications* and others)
- Priorities, opportunities, and challenges for integrating microorganisms into Earth system models for climate change prediction (2024, *mBio*)
- Microbial solutions for climate change require global partnership (2025, *mBio*)
- Microbes without borders: uniting societies for climate action (2025, *mBio* and other journals)
- Soil microbiome interventions for carbon sequestration and climate mitigation (2025, *mSystems*)
- Safeguarding microbial biodiversity: microbial conservation specialist group within the species survival commission of the International Union for Conservation of Nature (2025, *mSystems* and other journals)
- Launching the IUCN Microbial Conservation Specialist Group as a global safeguard for microbial biodiversity (2025, *Nature Microbiology*)
- The power of microbial life for the transformation towards a sustainable planet: key messages from the 2024 IUMS Congress in Florence, the city of the Renaissance (2025, *Microlife*)
- Microbial solutions for climate change: toward an economically resilient future (2025, report)
- Water, waterborne pathogens and public health: environmental drivers (2025, colloquium report)
- Microbial solutions for greenhouse gas emissions (in press, *Nature Reviews Microbiology*)
- Role of climate change on emerging and reemerging infectious diseases: from attribution to action in global health preparedness (in press, colloquium report)
